# Echocardiographic Evaluation of Atrial Remodelling for the Prognosis of Maintaining Sinus Rhythm after Electrical Cardioversion in Patients with Atrial Fibrillation

**DOI:** 10.3390/jcm12155158

**Published:** 2023-08-07

**Authors:** Paweł Wałek, Joanna Roskal-Wałek, Patryk Dłubis, Beata Wożakowska-Kapłon

**Affiliations:** 1 Collegium Medicum, Jan Kochanowski University, 25-317 Kielce, Poland; joanna.roskal.walek@wp.pl (J.R.-W.); patryk.dlubis@gmail.com (P.D.); bw.kaplon@poczta.onet.pl (B.W.-K.); 2 1st Clinic of Cardiology and Electrotherapy, Swietokrzyskie Cardiology Centre, 25-736 Kielce, Poland; 3 Ophthalmology Clinic, Voivodeship Regional Hospital, 25-736 Kielce, Poland

**Keywords:** atrial fibrillation, tissue Doppler, strain, strain rate, remodelling, echocardiography, cardioversion

## Abstract

Atrial fibrillation (AF) is the most common atrial tachyarrhythmia. One of the methods of AF treatment is direct current cardioversion (DCCV), but in the long-term follow-up we observe quite a high percentage of AF recurrences after this procedure. In order to assess the prognosis of DCCV effectiveness, we use clinical, biochemical and echocardiographic parameters. The objective of this review is to systematise the current knowledge on echocardiographic measurements in patients with persistent AF used to assess the progress of remodelling of the atrial wall, which affects the likelihood of maintaining sinus rhythm after DCCV. In this article, echocardiographic parameters for the evaluation of remodelling of the atrial wall are divided into groups referring to structural, mechanical, and electrical remodelling, as well as parameters for the evaluation of left ventricular filling pressure. The article aims to draw attention to the clinical value of echocardiographic measurements, which is the selection of patients who will maintain sinus rhythm after DCCV in the long-term follow-up, which will allow to avoid unnecessary risks associated with the procedure and enable the selection of the appropriate treatment strategy.

## 1. Introduction

Atrial fibrillation (AF) is the most common atrial tachyarrhythmia. It is estimated that up to 33.5 million patients may be affected worldwide, which does not include those with an asymptomatic form of the disease. The prevalence expected in 2030 is more than 15 million Europeans and 12.1 million Americans. It is estimated that 886,000 European citizens are first diagnosed with AF each year and that one in four adults over the age of 40 will experience AF in their lifetime [1,2,3,4,5].

The most serious complication of atrial fibrillation is arterial embolism of cardiac origin, including stroke and TIA (transient ischemic attack), with AF one of the main causes. AF is also associated with the progression of systolic and diastolic heart failure, decreased quality of life, and increased all-cause mortality [6,7,8]. Because AF does not always have obvious symptoms, diagnosis is often a challenge.

The mechanisms leading to the development of atrial fibrillation are not fully understood. The probable cause of AF is considered to be damage to the atrial muscle and the development of remodelling. Widely described modifiable and non-modifiable risk factors contribute significantly to this phenomenon. Persistent AF also leads to further damage and, in consequence, to progressive remodelling [9]. There are currently three main types of atrial remodelling: electrical, mechanical and structural. Electrical remodelling consists in shortening the action potential and refractory period of atrial cells, as well as changes in sarcolemmal sodium ion channels and gap junctions. Mechanical remodelling consists in the impairment and dyssynchrony of atrial wall muscle contraction caused mainly by the replacement of myocardial cells by fibroblasts and fibrous tissue. Structural remodelling is the enlargement of the atrial cavities. Changes in the structure and function of atrial wall cells may lead to the induction of atrial fibrillation. Each of these types of remodelling can be evaluated indirectly by means of echocardiography.

The main goal of AF treatment is to eliminate or reduce the symptoms associated with arrhythmia and to prevent thromboembolic complications [10]. Reduction of AF symptoms is achieved by restoring and maintaining sinus rhythm (SR) or by controlling the heart rate. For this purpose, pharmacotherapy, ablation, or pharmacological or electrical cardioversion (DCCV–direct current cardioversion) are used [10]. One of the most commonly applied methods of restoring SR is DCCV, which is characterised by easy performance and low cost, so it is frequently offered to patients with AF. The effectiveness of cardioversion in restoring SR in patients with persistent and paroxysmal AF is estimated at 75–88%, while SR can be maintained for 12 months in 70% of patients [11].

The objective of this review is to systematise the current knowledge on echocardiographic measurements in patients with persistent AF used to assess the progress of remodelling, which affects the likelihood of maintaining sinus rhythm after DCCV. In this article, echocardiographic parameters for the evaluation of remodelling are divided into groups referring to structural, mechanical, and electrical remodelling, as well as parameters for the evaluation of left ventricular filling pressure (Table 1). The article aims to draw attention to the clinical value of echocardiographic measurements, which is the selection of patients who will maintain sinus rhythm after DCCV in the long-term follow-up. Appropriate selection of patients with persistent AF for an appropriate treatment strategy will avoid unnecessary risks associated with the procedure.

## 2. Material and Methods

In March 2023, an extensive manual search was performed through the major electronic databases (PubMed, Google Scholar) in order to identify relevant studies published on atrial remodelling. The following search terms were used: “atrial fibrillation echocardiography”, “atrial fibrillation remodeling”, “atrial fibrillation cardioversion”, “atrial fibrillation atrial strain”, “atrial fibrillation emptying fraction”, “predictors for maintenance of sinus rhythm after cardioversion, echocardiography”, and “atrial fibrillation recurrence after successful cardioversion”, in different combinations. With regard to echocardiographic parameters with prognostic values in the prognosis of sinus rhythm failure after DCCV, original studies were selected and the latest review articles were included in the analysed reports. A total of 65 compatible research publications were identified and used to compile this review.

## 3. Structural Remodelling

Structural remodelling is the final stage of atrial remodelling resulting from preceding and accompanying electrical and mechanical remodelling. It is not confirmed whether AF is due to remodelling or remodelling is due to AF, although both mechanisms seem likely.

Parameters for structural remodelling evaluation are rather well described, and their correlation with the persistence of SR after DCCV has been thoroughly studied. The most commonly performed measurement is of the left atrium anteroposterior diameter (LAAP) during TTE (transthoracic echocardiogram). This is the least accurate parameter for evaluating the size of the left atrium (LA), since the enlargement of the LA is visible in the top–bottom measurement, so the real size of the LA is often underestimated. Despite numerous studies, the predictive value of this parameter remains unclear [12,13,14,15,16,17,18]. A more precise measurement of the LA size is the measurement of the left atrial volume (LAV) in the late systolic phase, indexed with the body surface as the left atrial volume index (LAVI). It has been shown that patients with recurrent AF have significantly increased LA sizes compared to those with SR, and that LA enlargement significantly increases the risk of AF recurrence [16,19,20,21]. LAVI measurements provide a reliable estimation of LA size. The introduction of LAVI improved the accuracy of prognosing AF recurrence after effective DCCV, both during AF and SR [15,22,23,24]. Compared to LAAP, LAVI has a significantly higher predictive value in terms of AF recurrence after DCCV [15].

In addition to evaluating the structure of the left atrium, assessment of the right atrium (RA) is also important. There are data indicating the superiority of the right atrial volume index (RAVI) over LAVI in evaluating the risk of AF recurrence after DCCV [25]. Atrial volume can also be measured with contrast computed tomography. The diagnostic value of such measurements, together with RAVI and LAVI for the evaluation of AF recurrence after PVI (pulmonary vein isolation), was studied [26]. The results of the study indicate that determining the ratio of RAVI to LAVI can be a useful and valuable indicator of the recurrence of arrhythmia [26].

## 4. Mechanical Remodelling

It is estimated that mechanical and structural remodelling of the LA is caused by excessive tension of the LA wall, which leads to the replacement of muscle fibres with connective tissue. Increased tension of the LA wall and a reduced number of muscle fibres lead to impaired contractility of the LA wall and the wall of its appendage, which in turn causes a dilation of the LA cavity and a decrease in myocardial velocity. The degree of mechanical remodelling is correlated with the duration of AF, as well as with the presence of mitral insufficiency [27]. An increasing number of researchers emphasise the superiority of parameters for measuring mechanical remodelling over parameters for measuring structural remodelling in evaluating the chances of maintaining SR in patients with AF [28,29,30,31]. These parameters can be evaluated after DCCV, but parameters during AF are also available for testing. The ability to assess the likelihood of maintaining SR prior to performing DCCV is extremely valuable, as it can provide a better assessment of the patient before qualifying for DCCV or an alternative treatment. In addition to the prognostic value in terms of AF recurrence after DCCV, some parameters measured during transoesophageal echocardiography (TEE), such as left atrial appendage wall motion velocity (LAAWMV) and peak atrial contraction strain (PACS), are also useful for evaluating the risk of thromboembolic complications in AF patients [32,33,34,35,36].

Assessment of mechanical remodelling of the LA can be performed using standard echocardiography, as well as new techniques, such as assessment of deformation of the LA wall during TEE or TTE. Standard echocardiographic measurements during TTE help in assessing mechanical remodelling by means of such parameters as LAEF (left atrial emptying fraction) and RAEF (right atrial emptying fraction), or AFc (left atrial fibrillatory contraction flow). During a TEE, it is possible to assess LAAFV (left atrial appendage flow velocity) using pulse Doppler imaging or LAAWMV (left atrial appendage wall motion velocity) using tissue Doppler imaging. New imaging techniques such as strain and strain rate allow us to more accurately assess the systolic function of the LA muscle. Strain and strain rate measurements can be performed by means of tissue Doppler imaging (TDI), but measurements taken with this technique are prone to error due to its angle dependence. Strain and strain rate measurements can also be performed by means of STE (speckle tracking echocardiography), which is free from angle dependence error but requires better visualisation than TDI. Using strain and strain rate techniques, we can assess the extensibility and contractility of the left atrial wall, as well as the dispersion of deformation of the LA walls and local motility disorders of the LA walls.

### 4.1. Mitral Inflow A-Wave Velocity

With the damage to the atria and the progression of remodelling, the mechanical function becomes impaired, which leads to a decrease in mitral inflow velocity. Cardioversion can restore the sinus rhythm, but not necessarily the mechanical function of the atria. The time to regain correct atrial mechanical function has been linked to several factors, one of which is the duration of AF [37]. Among the Doppler examination parameters evaluating the flow through the mitral valve, the A-wave velocity proved to be an independent risk factor for AF recurrence after DCCV in studies by Spiecker et al. and Grundvold et al. [38,39]. Using the functions of tissue Doppler imaging, the velocity of the A wave of mitral inflow can be assessed during TTE after successful cardioversion of atrial fibrillation. In a study by Spiecker et al., sinus rhythm was restored pharmacologically in 14% of patients and with DCCV in 77% of patients. Echocardiography was performed up to 4 h after cardioversion and then on the first, second and third day after restoring SR. In a multivariate regression analysis, the A-wave velocity of the mitral inflow measured one day after DCCV proved to be an independent predictor of AF recurrence. In subsequent studies, there was a gradual increase in the A-wave velocity from the day of cardioversion (mean 44 cm/s) until four weeks after it (72 cm/s). Patients whose AF duration was less than six weeks demonstrated a higher A-wave velocity after successful cardioversion. The E-wave mitral inflow velocity was comparable between patients with maintained SR and with recurrent AF [38]. Grundvold et al. also described the effect of low A-wave velocities of mitral flow on an increased risk of AF recurrence after cardioversion. Significantly lower mitral A-wave velocity (≤0.1 m/s) was reported in the 6-month follow-up in the group of patients with AF recurrence compared to the group where SR was maintained (≥0.45 m/s). There were no differences in atrial size or left ventricular function between the groups with recurrent AF and maintained SR [39].

### 4.2. Left and Right Atrial Emptying Fraction

**Figure 1:**
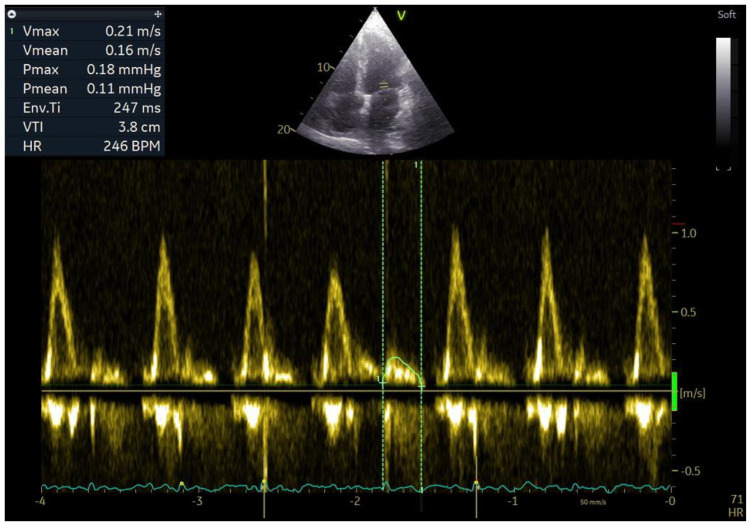
Left atrial fibrillatory contraction flow (Afc). Pulsed-wave Doppler of mitral filling in the apical four-chamber projection during atrial fibrillation. Velocity time integral (VTI) and AFc velocity measurement. AFc VTI 3.8 cm. AFc V 21 cm/s.

One of the parameters for evaluation of the mechanical function of the left and right atria is their emptying fraction. This is the ratio of the volume of blood ejected into the ventricle during atrial contraction to the volume of blood in the atrium before its contraction. It is calculated as follows: (LA maximum volume − LA minimum volume)/LA maximum volume × 100%. It allows for indirect assessment of the atrial contractility and the degree of progression of mechanical remodelling. Higher values of emptying fraction mean a larger volume of blood transported to the ventricles during ventricular diastole so that less blood remains in the atria. It is connected with a lower degree of stretching of the atria and, over a longer period, slower development of structural remodelling, which results mainly from mechanical remodelling. Mechanical remodelling has a negative effect on atrial contractility, and studies show that patients with maintained SR after DCCV had higher LAEF and RAEF than patients with recurrent AF, and that RAEF and LAEF had greater prognostic value in evaluating recurrent AF after DCCV than LAVI and RAVI [28]. Until recently, it was believed that atrial contractility during an episode of AF was disturbed to such an extent that it did not generate an LA emptying volume. Studies show that the emptying volume is still generated, despite contractility impairment during an episode of AF. In addition, measurement of the LA emptying fraction during AF has prognostic value in terms of the maintenance of SR after DCCV [40,41]. Kim et al. presented a method for evaluating the contractility of LA during an episode of AF, which consists in measuring the wave of mitral inflow to the LV that occurs between successive E waves of mitral inflow. This parameter was called Afc (atrial fibrillatory contraction flow), and it is assumed that the larger the Afc wave, the better the contractility of LA is preserved (Figure 1) [41]. Based on these analyses, the authors concluded that the velocity time integral of the Afc wave and its velocity predict AF recurrence after DCCV more accurately than LAVI. In our study, we found that patients who have a higher LAEF during AF also have a better prognosis in terms of maintaining sinus rhythm after DCCV than patients with a lower LAEF. In addition, the parameter that evaluates mechanical remodelling had greater prognostic value than parameters that evaluate structural remodelling [40].

### 4.3. Left Atrial Appendage Flow Velocity

Another echocardiographic parameter that allows us to assess the systolic function, and thus mechanical remodelling of the left atrium, is the left atrial appendage flow velocity (LAAFV), measured both during sinus rhythm and atrial fibrillation. The limitation of this parameter is that it must be measured during a TEE examination. In a healthy person, during the sinus rhythm, when the contractility of the left atrium appendage (LAA) is preserved, the top of the appendage during contraction almost completely closes. As a result of wall hardening, which occurs in remodelling, the contractility of the LAA decreases, which results in a decrease in the velocity of blood inflow and outflow. Measurement of LAAFV during TEE provides an indirect assessment of the systolic function of the LAA. The left atrium appendage has a characteristic emptying pattern during SR [42]. The highest emptying velocity occurs in the proximal part of the LAA: 50 to 83 cm/s in healthy individuals [43]. A velocity below 40 cm/s increases the risk of stroke and AF recurrence after successful DCCV. The higher the value, the higher the probability of maintaining SR after DCCV. In a study by Melduni et al., LAAFV was shown to have prognostic value in terms of assessment of sinus rhythm maintenance after DCCV, risk of stroke, and death in patients with atrial fibrillation. In the study, it was demonstrated that the risk of the above events increases with a decrease in LAAFV values [44]. Similarly, Antonelli et al. presented LAAFV as a predictor of the maintenance of SR after DCCV. In their study, LAAFV had greater predictive value in terms of the maintenance of SR after DCCV than the structural remodelling parameter LAAP [21].

### 4.4. Left and Right Atrial Wall Motion Velocity

Another parameter with high prognostic value for evaluation of the maintenance of SR after DCCV measured in TTE is the left and right atrial wall motion velocity—LAWMV and RAWMV. De Vos et al. evaluated the prognostic value of measurements of atrial wall motion velocity and duration of an AF cycle for evaluating the immediate success of DCCV and the maintenance of SR over one year after DCCV in patients with persistent AF. LAWMV and RAWMV were measured using TDI, and the measurements were performed retrospectively using the Q-analysis software on colour Doppler images. Measurements were performed during late diastole in the left lateral atrial wall just below the mitral valve ring and in the right lateral atrial wall just below the tricuspid valve ring. LAWMV did not have prognostic value for the immediate success of DCCV, but it was shown that patients with higher LAWMV values were more likely to maintain sinus rhythm after DCCV over a 12-month follow-up. In the case of RAWMV, it was shown that the higher the result of this measurement, the higher the chances of immediate success of DCCV and maintenance of sinus rhythm over a 12-month follow-up. In this study, the group of patients with maintained SR after DCCV did not differ in terms of structural remodelling parameters (LAAP, RAV, LAV) from the group of patients who had recurrent AF [45]. In addition, it was demonstrated that the longer the duration of AF, the lower the LAWMV values during atrial fibrillation, which suggests that the LAA wall motion velocity reflected mechanical remodelling [27]. A limitation of RAWMV and LAWMV measurements is that they are much more difficult in patients with high ventricular rhythm.

**Figure 2:**
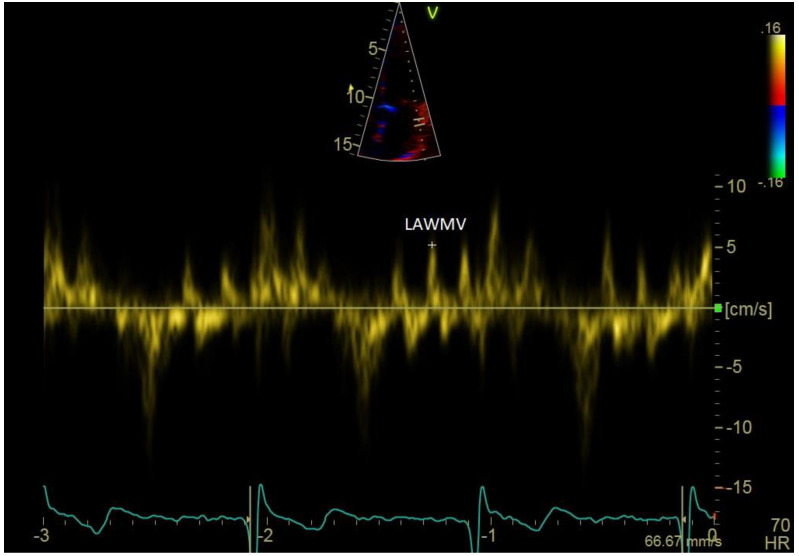
Left atrial wall motion velocity (LAWMV). LAWMV 5 cm/s. Pulsed-wave tissue Doppler in the apical four-chamber projection during atrial fibrillation.

Recently, left and right atrial wall motion velocity was proven to possess prognostic value in terms of predicting the maintenance of the sinus rhythm after electrical cardioversion performed due to persistent AF, in which velocity measurements were performed directly using tissue Doppler imaging (TDI) (Figure 2) [46]. In this study, in a multivariate regression analysis including clinical and echocardiographic factors, only LAWMV was a significant predictor of SR maintenance over 12 months. Direct measurement, compared to the Q-analysis from colour Doppler imaging used by De Vos et al., has some advantages. Direct measurement is much less time-consuming and does not require additional software. Additionally, in Q-analysis, the sampling rate of the velocity measurement is affected by the size of the colour Doppler image that is recorded. Direct measurement of the left atrial wall motion velocity by means of tissue Doppler imaging is performed in the same way as a’ and e’ measurements of the velocity of diastolic motion of the mitral valve ring, but the Doppler gate should be located approximately 10 mm below the ring. A clear advantage of using colour Doppler imaging and the Q-analysis software is the ability to perform retrospective measurements from recordings made earlier.

Assessment of the myocardial velocity in the atria appears to be a promising method for evaluating the likelihood of SR maintenance after DCCV in persistent atrial fibrillation, and hence it is important to further evaluate these parameters in a larger patient population.

### 4.5. Left Atrial Appendage Wall Motion Velocity

**Figure 3:**
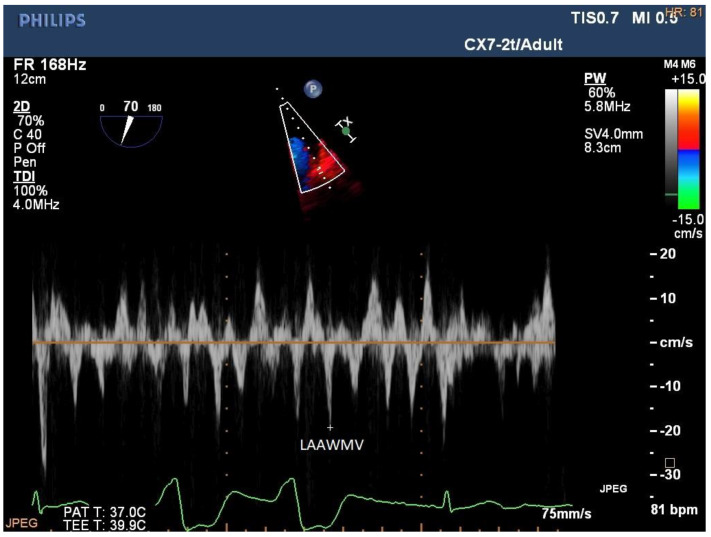
Left atrial appendage wall motion velocity (LAAWMV). LAAWMV 18 cm/s. Pulsed-wave tissue Doppler in two-chamber 60–90° projection in transoesophageal echocardiography during atrial fibrillation.

Another parameter in the direct evaluation of the mechanical function of the left atrial appendage is left atrial appendage wall motion velocity (LAAWMV) (Figure 3). Left atrial appendage wall motion velocity correlates with the degree of mechanical remodelling, which leads to a decrease in myocardial velocity. Patients whose LAAWMV is measured during AF have lower LAAWMV values than patients with sinus rhythm [47]. One of the advantages of LAAWMV over other markers of mechanical remodelling is that it can be measured before cardioversion during AF. There are two techniques available for evaluating LAA wall motion velocity: TDI (tissue Doppler imaging) and STE (speckle tracking echocardiography). Currently, STE is considered to be the preferred technique for evaluating myocardial velocity and deformity because it is free from angle dependence, which is the main limitation of TDI. The weakness of the STE technique is the need to obtain very good imaging quality, which is difficult in the case of thin walls of the LAA, whereas TDI does not have this limitation. Unfortunately, measurements of LAA wall motion velocity and deformation with STE can only be performed during TEE, while LAA wall motion velocity measurements with TDI can be performed using both TTE and TEE. The measurement of LAAWMV requires a break in the systolic function of the ventricle in order to visualise the systolic wave of the LAA wall motion. Rapid motion of the ventricles makes measurement difficult, but the stimulation of the vagus nerve that occurs during TEE slows down the heart rate, making reliable measurement of LAAWMV possible. In our study, LAAWMV had prognostic value both in terms of SR restoration after DCCV and SR maintenance over a 12-month follow-up after DCCV. Among the analysed echocardiographic parameters evaluated before DCCV during AF, only LAAWMV and E/e’ had prognostic value in terms of maintenance of SR after DCCV, both in multivariate models containing only echocardiographic parameters, including parameters for structural remodelling evaluation, and in the model with clinical parameters [31].

A recently discovered phenomenon is LAAWMV reduction in the lateral LAA wall compared to the LAA medial wall in patients with AF, while in patients without diagnosed AF, there is an opposite relation. The chances of identifying a patient with paroxysmal AF using this phenomenon was 22.14 (95% CI, 12.06–40.64; *p* < 0.001) [47].

### 4.6. Left Atrial Strain and Strain Rate

**Figure 4:**
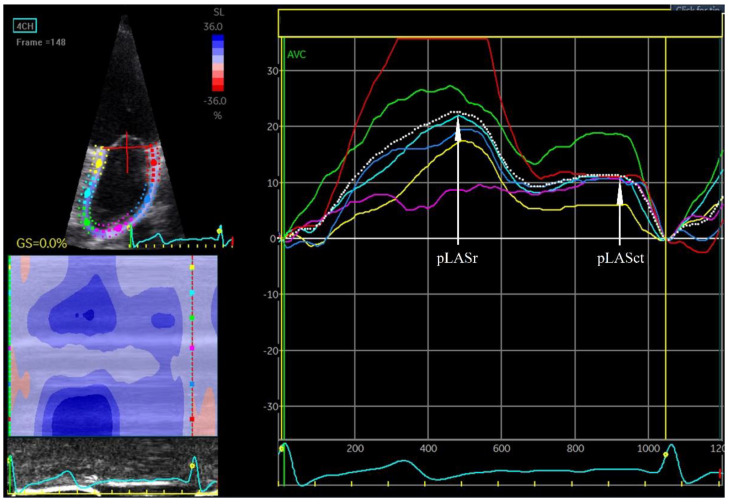
Left atrial strain. pLASr 22%, peak left atrial stain measured during the reservoir phase; pLASct 11%, peak left atrial stain measured during the contractile phase. The measurements are the average of the 6 assessed left atrial wall segments. The measurement was made in the apical four-chamber projection during sinus rhythm.

**Figure 5:**
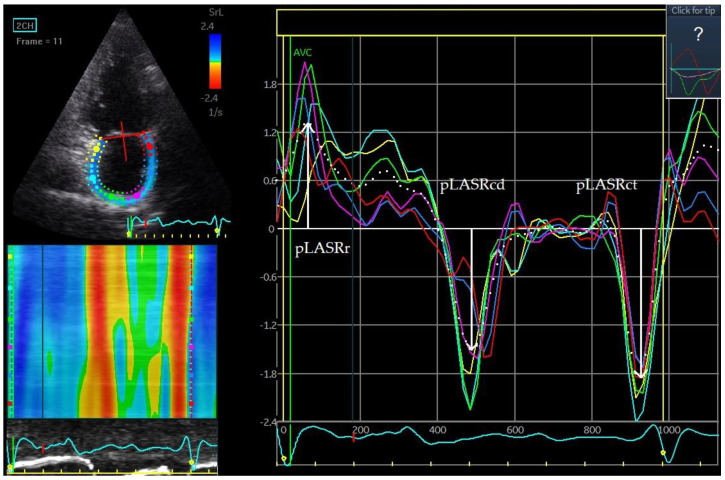
Left atrial strain rate. pLASRr 1.3 s^−1^ peak left atrial strain rate measured during the reservoir phase. pLASRcd −1.5 s^−1^, peak left atrial strain rate measured during the conduit phase. pLASRct −1.9 s^−1^, peak left atrial strain rate measured during the contractile phase. The measurements are the average of the 6 assessed left atrial wall segments. The measurement was made in the apical two-chamber projection during sinus rhythm.

A parameter called left atrial strain (LAS) is used to assess the degree of mechanical deformation of the myocardium. Progressive remodelling leading to atrial wall rigidity leads to a decrease in strain and strain rate during AF. Using the above-mentioned STE and TDI techniques, it is possible to assess the strain and strain rate during echocardiography [48]. An examination can be performed both during AF and after sinus rhythm restoration. In patients with sinus rhythm, the reservoir, conduit and contraction phases of LA can be evaluated (Figure 4 and Figure 5). In the reservoir phase, the susceptibility of the atrial wall to stretching is assessed. During the contraction phase—the ability of the atrial muscle to contract—and the conduit phase is the difference in measurement in the reservoir and contraction phases. During AF, only the conduit phase is evaluated, while the reservoir phase is a negative value of the conduit phase. Initially, strain and strain rate assessment were performed using TDI. One of the first studies of strain and strain rate using TDI to assess the prognosis of patients after DCCV in terms of maintenance of SR demonstrated the usefulness of these parameters in patients with persistent AF [29,49].

Decreased strain and strain rate were shown to be predictive of AF occurrence, especially in patients who have suffered a cryptogenic stroke [50,51]. Strain and strain rate are not only predictors of the occurrence of the first diagnosed AF, but can also be used to assess the probability of maintaining SR after a DCCV performed to treat AF. It was demonstrated that strain and strain rate measured the day after a successful DCCV are predictive of SR maintenance [52]. Moreover, the study revealed that strain and strain rate measured in the contraction phase have greater predictive value in terms of maintaining sinus rhythm than strain and strain rate assessed in the reservoir and conduit phases [52].

Strain measurements before cardioversion can predict the maintenance of sinus rhythm after DCCV. Morenzo-Ruiz et al. showed that the reservoir phase strain measured during AF before DCCV was predictive of SR maintenance over a 6-month follow-up, both in patients with persistent AF and long-standing persistent AF [53]. Shaikh et al. found that an increase in strain measured before DCCV and then after effective DCCV has prognostic value in terms of SR maintenance. The greater the increase in PALS after cardioversion relative to the measurement during AF, the greater the probability of maintaining SR over 6 months [54]. The left atrial conduit function can also be determined using the formula [(LV maximum − LV minimum) − (LA maximum − LA minimum) volume], expressed as % LV stroke volume. In studies evaluating LA conduit function, it was demonstrated that the parameter for LA conduit function evaluation is an independent risk factor of AF recurrence after DCCV [55,56].

Loss of coordinated atrial myocardial contraction is the result of mechanical remodelling and is described as dispersion. Strain evaluation also allows for the evaluation of LA wall contractility disorders such as dispersion and dyskinesia. Dispersion is defined as the standard deviation of the time to achieve maximum deformation in different atrial segments and indicates asynchronous contraction of individual LA walls [57,58,59]. Under normal conditions, all segments of the LA wall should reach maximum deformation at the same time of the heart cycle. Dell’era et al. demonstrated that asynchrony of the left atrial contraction has prognostic value for the maintenance of SR after DCCV [58]. Similarly, Doruchowska et al. proved that the dispersion of time to maximum strain is an important predictor of SR maintenance after DCCV [30]. In a study by Rondano et al., the standard deviation of the time to maximum deformation was inversely dependent on the value of the maximum deformation. This study also showed that asynchronous LA contraction is a predictor of AF recurrence in patients after an effective DCCV performed to treat AF [57].

**Figure 6:**
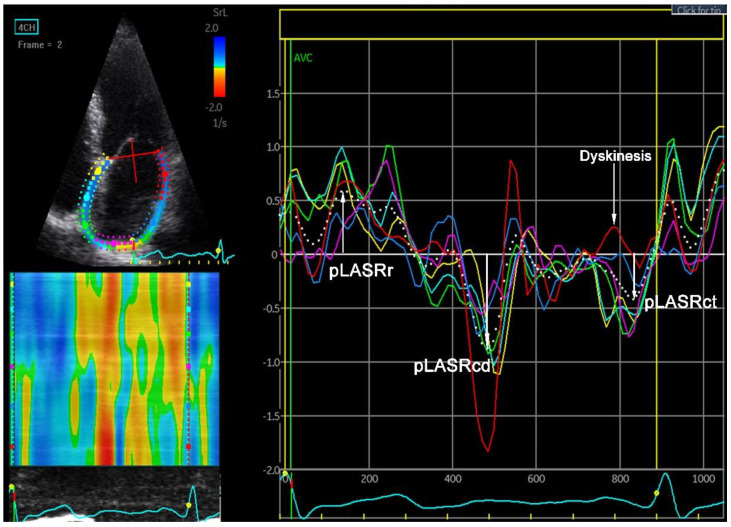
Left atrial wall dyskinesis. Stretching of one of the left atrial wall segments during the contractile phase. The phenomenon of left atrial wall dyskinesia is assessed during pLASRct; in the contractile phase, all segments of the left atrial wall should contract, while when dyskinesia occurs, one or several segments expand. The measurement was made in the apical four-chamber projection during sinus rhythm.

Left atrial wall dyskinesia is a new indicator that can be used to assess the likelihood of maintaining SR after DCCV in persistent AF. Left atrial wall dyskinesia means that certain segments of the atrial wall expand rather than contract during the contraction phase. In measurements of the LA strain rate in the contraction phase, dyskinetic segments demonstrate positive strain rate values, which indicates that they are stretching relative to adjacent segments that have negative strain rate values, which indicates that they are contracting (Figure 6). This phenomenon is analogous to dyskinesia of segments of the left ventricle walls. A study evaluating strain rate the day after effective DCCV showed that the appearance of dyskinetic LA wall segments increases the risk of AF recurrence after effective DCCV over a 12-month follow-up [60].

## 5. Electrical Remodelling

**Figure 7:**
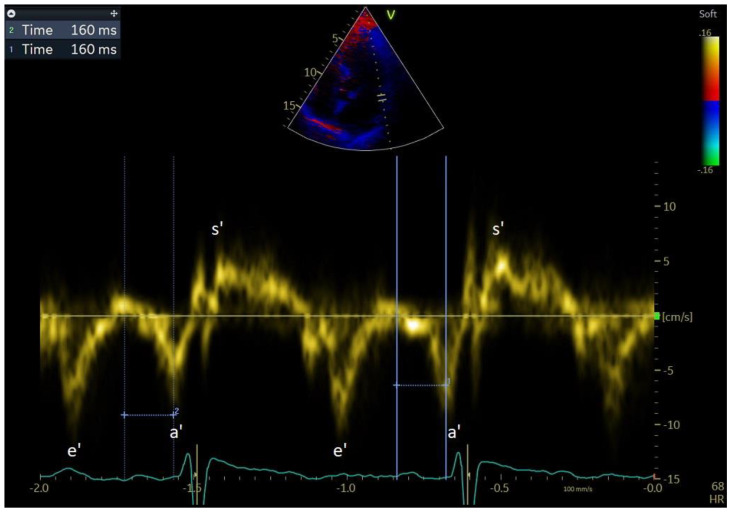
Total atrial conduction time (TACT). PA-TDI 160 ms. PA-TDI is the time interval between the onset of the P wave in electrocardiogram in lead I or II and the peak of the a’ wave on the atrial tissue Doppler velocity curve from the lateral left atrial wall. Pulsed-wave tissue Doppler of mitral annulus velocity in the apical four-chamber projection during sinus rhythm. a’, late lateral mitral annulus velocity; e’, early lateral mitral annulus velocity; s’, systolic mitral annulus velocity.

The type of remodelling that occurs first, and is also the least visible and difficult to visualise with echocardiography, is electrical remodelling. Electrical remodelling of the atrial walls is an expression of disorders in intercellular connections, change in ion channels, progressive LA wall fibrosis, and abnormalities in the conduction of electrical stimuli between the cells of the atrial wall, which results in slowing down of electrical stimuli conduction and the development of micro-re-entry substrate [61,62]. A lack of balance between collagen synthesis and degradation leads to myolysis and the development of atrial fibrosis, which in turn affects the electromechanical function of the LA, manifested by a longer atrial conduction time [63,64]. Currently, the only echocardiographic parameter that depends on electrical remodelling but also on structural remodelling is total atrial conduction time (TACT) (Figure 7). TACT is affected by both slowing down of the electrical stimuli conduction in the atrial wall, i.e., electrical remodelling, and by the length of the path that electrical stimuli must travel, i.e., enlargement of the right and left atrial cavity, which is an expression of structural remodelling. The delay between the P wave in the electrocardiogram from lead I or II and mechanical LA activation measured by tissue Doppler echocardiography, called PA-TDI, provides a reliable estimate of total atrial activation time, reflecting the degree of atrial fibrosis in biopsy samples [65]. In a study by Leung et al., PA-TDI was shown to be significantly higher in patients with AF compared to those without AF [66]. TACT prolongation reflects the slowing down of conduction and dilation of the atria and identifies individuals prone to developing atrial fibrillation [67,68].

TACT was demonstrated to be predictive of AF recurrence after DCCV. Müller et al. showed that patients with prolonged TACT have a higher risk of early AF recurrence after DCCV. Using a multivariate regression model, they demonstrated the superiority of this parameter over a parameter for evaluation of structural remodelling, but they did not assess echocardiographic parameters representing the progression of mechanical remodelling [69]. The prognostic value of TACT for the maintenance of SR after DCCV in AF was also investigated in long-term studies. Using the same method to evaluate PA-TDI as Muller et al., Maffe et al. evaluated the prognostic value of TACT in patients with persistent AF undergoing DCCV at the annual SR maintenance assessment. Maffe et al. also confirmed the prognostic value of TACT, which was the best predictor of the chances of maintaining SR after DCCV in the studied population. The analysed parameters also included parameters for evaluating structural remodelling (LAVI, RAVI) and mechanical remodelling (a’ wave, LAEF). The analysed echocardiographic parameters did not include strain or strain rate [70]. Karantoumanis et al. also showed the predictive value of TACT in terms of maintenance of SR after DCCV. In a multivariate regression model and ROC curve analysis, they proved the superiority of TACT over the parameters for evaluating structural remodelling (LAVI, RAVI) and mechanical remodelling (LAEF). The authors also evaluated left and right atrial strain defined as “left and right atrial peak longitudinal strain during ventricular systole” and the left atrial strain rate as “left atrial peak longitudinal strain rate during ventricular systole.” These parameters did not differ between the group of patients with maintained sinus rhythm and the group of patients with recurrent AF. The study also analysed left atrial longitudinal strain, but it was not precisely described how this was measured, and despite obtaining statistical significance in the studied group, it was not further analysed in multivariate regression models [71].

Evaluation of electrical remodelling seems to be a very promising method for evaluating the prognosis of AF recurrence after DCCV in patients with persistent AF, but echocardiography does not currently seem to be a good tool for evaluating these types of changes in the left or right atrial wall.

## 6. Left Ventricular Filling Pressure

A separate parameter that does not concern the assessment of atrial remodelling but is an important predictor of AF recurrence after electrical cardioversion in persistent atrial fibrillation is left ventricular filling pressure (LVFP), which assesses left ventricular diastolic dysfunction. Left ventricular filling pressure is estimated during sinus rhythm by measuring mitral valve inflow waves E and A, the e’ wave of the diastolic motion of the mitral valve ring, and the mitral inflow E wave deceleration time (DT).

Assessment of the filling pressure is also possible during AF, but estimation of the LV filling pressure is difficult because the lack of the A wave in the mitral inflow and the high variability of E and e’ waves in successive heart cycles negatively affects the accuracy of the estimate and the variability of subsequent measurements.

In studies evaluating left ventricular filling pressure based on the E/e’ ratio, it was demonstrated that the LV filling pressure assessed during persistent AF and after successful DCCV is a predictor of SR maintenance after DCCV [72,73,74]. In a study by Chung et al., an increased E/e’ ratio significantly correlated with AF recurrence after cardioversion [71]. In a study by Fornengo et al., DT <150 ms (E-wave deceleration time), septal e’-wave <8 cm/s and septal E/e’ ratio ≥11 were considered as the cut-off point for increased LV filling pressure [73]. Similarly, in our study, we showed that elevated values of E/a and E/e’ are predictors of AF recurrence after DCCV in patients with normal systolic function of the LV muscle. In this study, in a multivariate regression model, parameters evaluating the left ventricular filling pressure had greater predictive value for SR maintenance after DCCV than parameters evaluating structural remodelling (LAVI) or mechanical remodelling LA (LAEF). The study did not measure mechanical remodelling parameters using the strain or strain rate methods [75].

## 7. Summary

Although ablation is becoming an increasingly common and effective method of treating patients with both paroxysmal and persistent AF, cardioversion remains one of the most frequently performed procedures in cardiology departments [76,77]. As there are no fully effective and safe antiarrhythmic drugs, the risk of recurrence of atrial fibrillation after DCCV remains high. The use of echocardiographic parameters to assess the prognosis for AF recurrence after DCCV has clear advantages in terms of availability, repeatability and better prognostic value for the maintenance of sinus rhythm after DCCV than clinical or biochemical parameters. Echocardiography remains the method of choice for evaluating the structure and function of the atria and ventricles compared to MRI and computed tomography, due to its greater availability and safety. Therefore, it is important to search for new, improved echocardiographic parameters that correlate with the progression of remodelling and allow for a more accurate assessment of the risk of AF recurrence after DCCV. Table 2 shows the optimal cut-off, area under the curve, sensitivity and specificity of the presented echocardiographic parameters for estimating the risk of recurrence of AF after DCCV. Based on this review of the available literature, it can be concluded that echocardiographic parameters evaluating mechanical remodelling of the left atrium have greater prognostic value than parameters evaluating structural remodelling in terms of prognosis of maintenance of sinus rhythm after DCCV in persistent and paroxysmal AF, and electrical remodelling can only be indirectly assessed by echocardiographic examination. Most of the echocardiographic parameters presented in this article have not yet been introduced into the daily clinical practice of qualifying patients with AF for treatment. This is because advanced software is required, some of the parameters have low repeatability, and their measurement can be time-consuming. However, some of the presented echocardiographic parameters can be performed without the involvement of additional time or advanced software, such as AFc or LAWMV. From a practical point of view, time invested in the assessment of a patient with AF allows us to more accurately qualify them for individual forms of therapy, such as pharmacotherapy, cardioversion or ablation. As ablation is a rapidly developing form of AF therapy, the above echocardiographic parameters should be studied further in terms of their prognostic value for maintaining sinus rhythm after ablation, pulmonary vein isolation or possible qualification of patients for expanded forms of standard ablation [78,79].

## Figures and Tables

**Table 1:** Echocardiographic parameters evaluating atrial remodelling.

Structural Remodelling	Mechanical Remodelling	Electrical Remodelling
1. Left atrial anteroposterior diameter (LAAP)	1. Mitral inflow A-wave velocity	1. Total atrial conduction time (TACT) *
2. Left atrial volume (LAV) in the late systolic phase and left atrial volume in the late systolic phase indexed with body surface–left atrial volume index (LAVI).	2. Left and right atrial emptying fraction (LAEF and RAEF)	
3. Right atrial volume (RAV) in the late systolic phase and right atrial volume in the late systolic phase indexed with body surface–right atrial volume index (RAVI).	3. Measurement of mitral inflow velocity in the LV (Afc)	
4. Total atrial conduction time (TACT) *	4. Inflow and outflow velocity in the appendage of the LA–left atrial appendage flow velocity (LAAFV)	
	5. Left and right atrial wall motion velocity (LAWMV and RAWMV)	
	6. Left atrial appendage wall motion velocity (LAAWMV)	
	7. Left atrial strain and strain rate	

* Depends on the size (structural remodelling) and the conduction velocity of the electrical excitation (electrical remodelling).

**Table 2:** Prognostic value of echocardiographic parameters in terms of maintenance of sinus rhythm/atrial fibrillation recurrence * after electrical cardioversion.

Parameter	Number of Patients	Paroxysmal/Persistent AF	Optimal Cut-Off	AUC	Sensitivity (%)	Specificity (%)	Follow-Up (months)
LAVI * [15]	76	No distinction	31 mL/m^2^	0.78	71.2	78.3	>12 months
LAVI [28]	95	Persistent	48 mL/m^2^	0.63	52	79	6
RAVI [28]	95	Persistent	43 mL/m^2^	0.76	72	82	6
Mitral inflow A-wave velocity * [38]	112	No distinction	52 cm/s	0.710	91	43	1
LAEF in AF [28]	95	Persistent	42%	0.89	73	100	6
RAEF in AF [28]	95	Persistent	52%	0.92	88	89	6
LAEF in AF [40]	146	Persistent	23.9%	0.680	83.6	51.2	12
AFc * VTI [41]	137	Persistent	3.1 cm	0.962	97	75.7	5
AFc * velocity [41]	137	Persistent	32 cm/s	0.857	96.8	94.1	5
LAAFV in AF [21]	186	>48 h <1 year of AF	40 cm/s	0.700	56	80	12
RAWMV in AF [45]	133	Persistent	No data	0.67	No data	No data	12
LAWMV in AF [45]	133	Persistent	No data	0.66	No data	No data	12
LAWMV in AF [46]	126	Persistent	3 cm/s	0.738	92.7	49.3	12
LAAWMV in AF [31]	121	Persistent	7.16 cm/s	0.738	76.5	70	12
pLASR (inferior wall) in AF [29]	65	No distinction. ≤3 months	1.8 sek^−1^	0.878	92	78	3
pLAS (septal) in AF [29]	65	No distinction. ≤3 months	22%	0.852	77	86	3
pLASR cd in AF (basal left) [49]	52	No distinction. <1 year	2.18 s^−1^	0.860	83.9	64.3	1
pLAS ct 4c in SR [52]	89	Persistent	No data	0.765	No data	No data	12
pLAS r *^,1^ in AF [53]	131	Persistent	10.75%	0.954	85	99	6
TP-SD Left atrial asynchrony [57]	130	Persistent	15%	No data	82.4	35	12
dTPLS in SR [30]	80 (61 restored SR)	Persistent	128 ms	0.660	57	83	6
Left atrial wall dyskinesia * in SR [60]	89	Persistent	Binary variable	0.71	59.57	82.5	12
TACT * in SR [69]	54	Persistent	152 ms	0.990	87	100	7 days
TACT * in SR [70]	104	Persistent	152 ms	0.923	91	87	12
TACT * in SR [71]	60	39 patients paroxysmal AF and 21 with persistent AF	125.8 ms	0.989	98	100	12
E/e’ in AF [40]	146	Persistent	8.7	0.645	73.8	55.4	12
E/e’ * in AF [72]	66	Persistent	9.15	0.780	75	73.1	>12 months
E/e’ * in AF [74]	175	Persistent	11	0.660	No data	85	3
E/e’ in SR [75]	117	Persistent	9.17	0.726	72.1	74.1	12
E/A in SR [75]	117	Persistent	2.2	0.726	73.12	73.21	12

* Parameters assessing the recurrence of atrial fibrillation after electrical cardioversion. ^1^ pLAS (GPALS)—mean of strain results obtained from apical four and two chamber views. 4c, four-chamber view; AF, atrial fibrillation; AFc, left atrial fibrillatory contraction flow; AUC, area under the curve; cd, conduit phase; ct, contraction phase; dTPLS, dispersion of time to peak longitudinal strain; LAAFV, left atrial appendage flow velocity; LAAWMV, left atrial appendage wall motion velocity; LAEF, left atrial emptying fraction; LAVI, left atrial volume index; LAWMV, left atrial wall motion velocity; pLAS, peak left atrial strain; pLASR, peak left atrial strain rate; r, reservoir phase; RAEF, right atrial emptying fraction; RAVI, right atrial volume index; RAWMV, right atrial wall motion velocity; SR, sinus rhythm; TACT, total atrial conduction time; VTI, velocity time integral.

## Data Availability

Not applicable.
